# The significance of cardiac inflammatory protocol of FDG PET-CT in the diagnosis and response assessment of tuberculous pericarditis: A case report

**DOI:** 10.22038/aojnmb.2025.80912.1580

**Published:** 2025

**Authors:** Kabilash Dhayalan, Harish Goyal, Dhanapathi Halanaik, Shaheer Ahamed

**Affiliations:** 1 Department of Nuclear Medicine, Jawaharlal Institute of Postgraduate Medical Education and Research, Puducherry, India; 2 Department of Cardiology, Jawaharlal Institute of Postgraduate Medical Education and Research, Puducherry, India

**Keywords:** Tuberculous pericarditis, ^18^FDG PET-CT, Cardiac inflammation protocol, Pericardial diseases, Response assessment

## Abstract

Tuberculous pericarditis (TBP) is a rare but potentially life-threatening manifestation of tuberculosis, often presenting with nonspecific symptoms and varied clinical features. The disease is characterised by inflammation of the pericardium due to mycobacterium tuberculosis, leading to complications such as effusion, tamponade, and, in chronic cases, constrictive pericarditis. TBP is associated with high mortality, particularly if not promptly diagnosed and treated. ^18^F-fluorodeoxyglucose positron emission tomography-computed tomography (^18^FDG PET-CT) has proven invaluable in diagnosing and managing TBP. This imaging modality allows for precise inflammatory activity localisation and differentiates TBP from other causes of pericardial disease. Additionally, the cardiac inflammation protocol of ^18^FDG PET-CT enhances imaging accuracy by suppressing the normal physiological FDG uptake in the myocardium. In this case report, we highlight the pivotal role of the cardiac inflammation protocol of ^18^FDG PET-CT in both the initial diagnosis and subsequent response assessment of TBP, underscoring its importance in clinical practice.

## Introduction

 Tuberculous pericarditis (TBP) is the most common aetiology of constrictive pericarditis in the Indian population. Constrictive pericarditis commonly presents with symptoms related to volume overload, such as weight gain and swelling, or may be related to decrease cardiac output, like progressive fatigue and dyspnea on exertion (1). Additionally, an evening rise in temperature may be present in tuberculosis cases. Electrocardiogram and echocardio-graphy help arrive at a diagnosis, but the aetiology is often missed. It is of utmost importance to find the aetiology to guide proper management. The role of ^18^FDG PET-CT in the response assessment of skeletal tuberculosis is already known (2). Certain case studies have already been published showing the role of ^18^F-FDG PET-CT in diagnosing tuberculous pericarditis (3). Case reports have already been published showing the role of response assessment in treating tuberculous pericarditis (4, 5). In this case report, we highlight the utility of the cardiac inflammatory protocol of ^18^FDG PET-CT in the diagnosis and treatment of tuberculous pericarditis and it’s follow-up.

## Cases

 A 38-year-old female presented with a one-month history of shortness of breath, along with an associated evening rise in temperature of up to 100 °F. Physical examination revealed distant heart sounds and jugular venous distension. Initial laboratory investigations showed an elevated erythrocyte sedimentation rate (ESR) at 48 mm/hr. ECG showed T wave inversion in the inferior and lateral leads, and echocardio-graphy showed grade II diastolic dysfunction, concentric LV hypertrophy, and global hypokinesia. The LV cavity was not dilated. The calculated ejection fraction was on the lower side at 35%. A cardiac catheterisation study was done, which showed elevated and equal LV and RV end-diastolic pressure, elevated RA mean pressure and elevated LA mean pressure at 20 mm of Hg, prominent y descent, dip and plateau/ square root pattern LV and RV diastolic pressures. A diagnosis of constrictive pericarditis was considered, and FDG PET-CT was done to identify the aetiology. 

**Figure 1:**
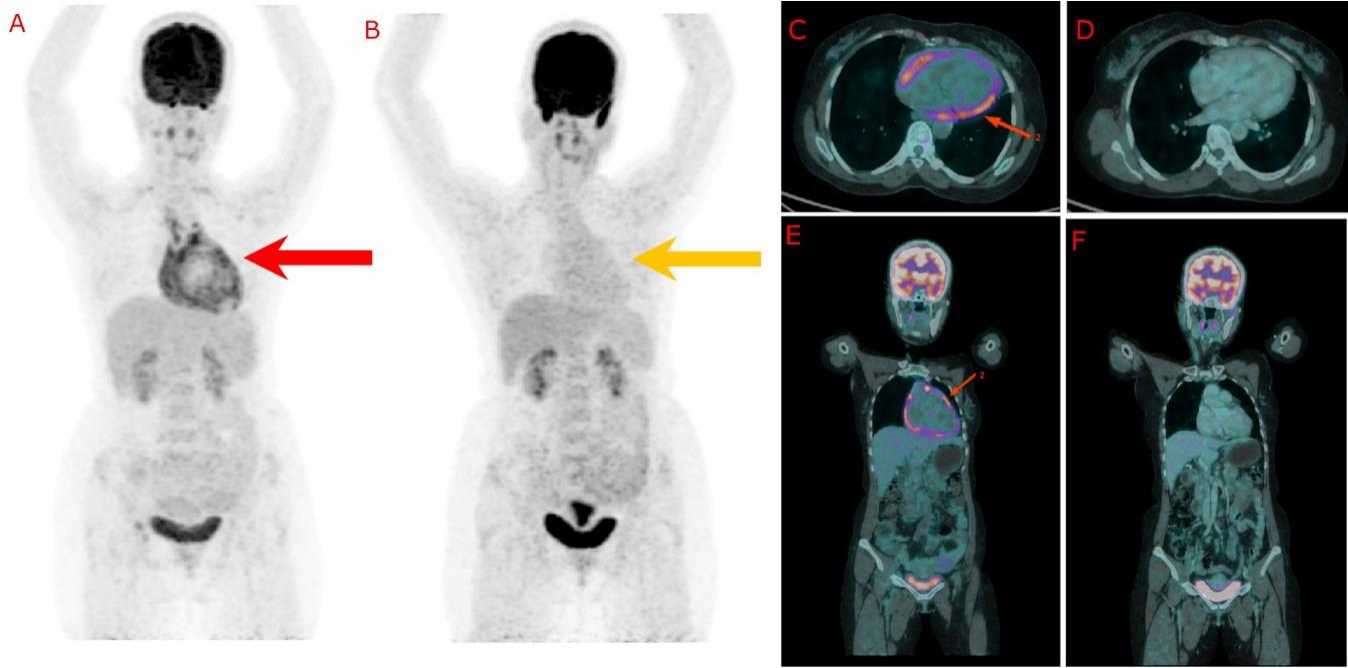
**A**: MIP whole body image before ATT showing pericardial FDG uptake (**red arrow**), **B**: MIP whole body image after ATT showing resolution of FDG uptake (**yellow arrow**), **C**: Fused axial image of the patient before ATT showing pericardial FDG uptake (**red arrow**) **D**: Fused axial image of the patient after ATT **E**: Fused coronal image of the patient showing pericardial FDG uptake (**red arrow**) **F**: Fused coronal image of the patient after ATT showing resolution of FDG uptake. MIP - Maximum intensity projection. ATT - Anti-tubercular therapy, FDG - Fluorodeoxyglucose

 The FDG PET-CT was done under cardiac inflammation protocol using combined prolonged fasting for 18 hours, a Fat-enriched carbohydrate-poor diet for 3 days before the study, and 50 IU/kg intravenous heparin 15 minutes before the FDG injection. The images are given in Figure 1. The scan revealed good myocardial suppression, intense fluorodeoxy-glucose uptake surrounding the pericardium, and focal uptake in the basal interventricular septal region, consistent with active inflammation. Additionally, increased FDG uptake was noted in bilateral cervical, mediastinal, right pelvic, and right inguinal lymph nodes, suggesting systemic involvement. 

 The findings supported the diagnosis of tuberculous pericarditis. Endomyocardial biopsy was done from the basal interventricular septum, and cytological analysis was done from the right cervical lymph node. None of them showed features suggestive of tuberculosis or granulomatous inflammation. Given the endemicity of tuberculosis in India, She was started empirically on an antitubercular regimen (isoniazid, rifampicin, ethambutol, and pyrazinamide) under category II under NTEP and completed a 6-month course. Follow-up FDG PET-CT at 1 month after the completion of treatment demonstrated a complete absence of pericardial FDG uptake, indicating a positive response to treatment. Clinical symptoms also improved, and inflammatory markers normalised.

## Discussion

Tuberculous pericarditis is a common cause of constrictive pericarditis with significant mortality. It is a treatable disease if diagnosed early. In the recent ESC guidelines (6) for managing pericardial diseases, ^18^FDG PET-CT was indicated in selected cases, such as patients with known malignant conditions and a possible non-invasive diagnosis of tuberculous pericarditis (7). In one retrospective study (3), it has been shown that ^18^FDG PET-CT has a role in guiding the pericardial and extracardiac tissue biopsy to arrive at a tissue diagnosis. The limitation of ^18^FDG PET-CT in the later indication is its poor ability to differentiate between physiologic and pathologic FDG uptake (8). Various patient preparation protocols (9) have been investigated and validated in cardiac sarcoidosis, either alone or with good results. The goal of these preparation protocols is to suppress the physiologic ^18^FDG uptake. Our case report illustrates a very good myocardial suppression, aiding in adequately visualising the pericardial ^18^FDG uptake. The stated results have been achieved only by proper patient preparation. The other differentials of this case include idiopathic pericarditis, viral pericarditis, connective tissue disorders and neoplastic causes (6). Since the patient responded well to anti-tubercular therapy, this is considered as tuberculous pericarditis.

## Conclusion

 This case report emphasises the valuable role of the cardiac inflammatory protocol of ^18^FDG PET-CT in the diagnosis and response assessment of tuberculous pericarditis. The imaging modality provides useful information on disease extent, aiding early diagnosis and monitoring treatment response. As TBP can have life-threatening complications, timely intervention guided by ^18^FDG PET-CT findings can significantly impact patient outcomes. 

 Further research and more extensive studies are warranted to establish the standardised use of cardiac inflammation protocol for ^18^FDG PET-CT in managing tuberculous pericarditis.
